# Improved CD4 T cell profile in HIV-infected subjects on maraviroc-containing therapy is associated with better responsiveness to HBV vaccination

**DOI:** 10.1186/s12967-018-1617-1

**Published:** 2018-08-29

**Authors:** Inés Herrero-Fernández, Isaac Rosado-Sánchez, Miguel Genebat, Laura Tarancón-Díez, María Mar Rodríguez-Méndez, María Mar Pozo-Balado, Carmen Lozano, Ezequiel Ruiz-Mateos, Manuel Leal, Yolanda M. Pacheco

**Affiliations:** 1 Institute of Biomedicine of Seville (IBiS), Virgen del Rocío University Hospital (HUVR)/CSIC/University of Seville, Avda. Manuel Siurot s/n, PC 41013 Seville, Spain; 2 Microbiology Service, Virgen del Rocío University Hospital (HUVR), Seville, Spain; 3 Internal Medicine Service, Hospital Viamed Santa Ángela de la Cruz, Seville, Spain

**Keywords:** Maraviroc **(**MVC), CD4 T-cell, Ki67, Activation, Treg, hsCRP, Inflammation, HBV vaccine, Dendritic cells (DC), Recent thymic emigrants (RTE)

## Abstract

**Background:**

Maraviroc-containing combined antiretroviral therapy (MVC-cART) improved the response to the hepatitis B virus (HBV) vaccine in HIV-infected subjects younger than 50 years old. We aimed here to explore the effect of this antiretroviral therapy on different immunological parameters that could account for this effect.

**Methods:**

We analysed baseline samples of vaccinated subjects under 50 years old (n = 41). We characterized the maturational subsets and the expression of activation, senescence and prone-to-apoptosis markers on CD4 T-cells; we also quantified T-regulatory cells (Treg) and dendritic cell (DC) subsets. We used binary logistic regression to evaluate the immunological impact of MVC-cART, correlation with MVC exposure and linear regression for association with the magnitude of the HBV vaccine response.

**Results:**

HIV-infected subjects on MVC-cART prior to vaccination showed increased recent thymic emigrants levels and reduced myeloid-DC levels. A longer exposure to MVC-cART was associated with lower frequencies of Tregs and activated and proliferating CD4 T-cells. Furthermore, the frequencies of activated and proliferating CD4 T-cells were inversely associated with the magnitude of the HBV vaccine response.

**Conclusion:**

The beneficial effect of MVC-cART in the HBV vaccine response in subjects below 50 years old could be partially mediated by its reducing effect on the frequencies of activated and proliferating CD4 T-cells prior to vaccination.

**Electronic supplementary material:**

The online version of this article (10.1186/s12967-018-1617-1) contains supplementary material, which is available to authorized users.

## Background

Human immunodeficiency virus (HIV)-infected subjects are at high risk for hepatitis B virus (HBV) infection and progression of severe, life-threatening hepatic complications, such as cirrhosis and hepatocellular carcinoma [1, 2]. To prevent the associated morbimortality, worldwide current guidelines recommend vaccination against HBV in all HIV-infected subjects susceptible to be coinfected by HBV, but the response rates are lower than in HIV-uninfected subjects [reviewed in 3].

The best-known predictors of vaccine efficacy are an undetectable viral load and CD4 T-cell counts above 350 cells/mm^3^ [3]. Thus, it is well assumed that successful combined antiretroviral therapy (cART) favours the vaccine response; however, the influence of the type of antiretroviral treatment has been scarcely explored until now. In this line, it was first described that maraviroc (MVC), a CCR5 antagonist, enhanced meningococcal neo-immunization and accelerated the response to tetanus boost [4]. More recently, we also reported that MVC-containing cART (MVC-cART) was associated with a better response against the HBV vaccine, at least in subjects younger than 50 years old [5]. Nevertheless, the potential underlying mechanisms were unaddressed.

Different antiretroviral combinations including MVC have comparatively proved their beneficial effects on the levels of inflammatory biomarkers [6, 7] and the T-cell immunophenotype [8]. In two clinical trials, an improvement of duodenal immunity and a reduction in bone loss has been associated with such combinations [9, 10]. Furthermore, MVC monotherapy also reduced the frequency of regulatory T-cells (Treg) in antiretroviral-naïve subjects [11], even improving the distribution of Treg subsets [12]. This could be relevant since we observed that Treg cells negatively impacted the HBV vaccine responsiveness in a previous cohort [13]. It is possible that MVC could enhance different functions required to mount an effective response following HBV vaccination, including antigen-presentation, T-cell help, regulatory T-cell suppression and B cell functions [14, 15].

In the present study, we aimed to explore the potential effect of MVC-cART in different parameters related to inflammation, T-cell function and dendritic cell subsets that could account for its effect on the HBV vaccine response; to this aim, we studied the same cohort of vaccinated subjects that had revealed a positive effect of such MVC-cART.

## Methods

### Study design, patients and samples

The vaccination protocol has been reported elsewhere [5]. Briefly, HIV-infected subjects from the Virgen del Rocío University Hospital were consecutively vaccinated against HBV. These subjects (a) were on suppressive cART (at least in the last 6 months), (b) had CD4 T-cell counts of > 300 cells/µl, (c) had negative serology for HBsAg and anti-HBc and (d) had anti-HBs titers of ≤ 10 mIU/ml. The vaccination protocol consisted of 3 intramuscular double doses (40 µg) of the recombinant Engerix-B vaccine (GlaxoSmithKline, Brentford, United Kingdom) at 0, 1, and 3 months. The vaccine response was measured 6 months after the first dose. A group of subjects was simultaneously vaccinated at 0 and 6 months against hepatitis A virus (HAV) (simultaneous HAV vaccination) with two intramuscular doses of the vaccine Havrix-1440 (GlaxoSmithKline, Brentford, United Kingdom). This subgroup of subjects had a previous negative serology for HAV. Fresh blood samples were collected at baseline, just before the administration of the first vaccine dose. All patients gave informed consent to enter the study, which was approved by the Ethic Committee of our Hospital. We restricted the present analyses to subjects younger than 50 years old (n = 41) from the total vaccinated population because the beneficial effect of MVC-cART on the vaccine response was observed in this population [5].

### Laboratory measurements

Absolute numbers of CD4 and CD8 T cells were determined with an Epics XL-MCL flow cytometer (Beckman-Coulter). Plasma HIV-1 RNA levels were measured using quantitative PCR (Cobas Ampliprep/Cobas TaqMan HIV-1 test; Roche Molecular Systems, Basel, Switzerland) with a detection limit of 20 HIV-RNA copies/ml. Plasma samples were tested for HBV-related markers (HBsAg, anti-HBs, and anti-HBc) using an HBV enzyme-linked immunosorbent assay (ELISA; Siemens Healthcare Diagnosis, Malvern, PA). Qualitative PCR amplification was used for plasma hepatitis C virus (HCV) amplification (Cobas Amplicor; Roche Diagnosis, Mannheim, Germany) with a detection limit of 15 IU/ml. The highly sensitive C-reactive protein (hsCRP) levels were determined with an immunoturbidimetric serum assay using a Cobas 701 (Roche Diagnostics, Mannheim, Germany).

### Flow cytometry

Peripheral blood mononuclear cells (PBMCs) were isolated from fresh blood before the first dose of vaccine and cryopreserved. For the immunophenotyping of cellular subsets, PBMCs were thawed and immediately stained with the following surface antibodies: anti-CD31 PE-CF594, anti-CD56 BV510, anti-CD25 BV605, anti-CD45RA BV650, anti-CD4 BV786, anti-CD3 APC-H7, Lin2 FITC (anti-CD3, anti-CD19, anti-CD20, anti-CD14 and anti-CD56), anti-CD11c BV650, and anti-HLA-DR BV711 (BD Biosciences, USA); anti-CD39 FITC, anti-CD57 PE-Cy7, anti-HLA-DR BV570, anti-CD95 BV711, and anti-CD27 AF700 (BioLegend, USA); and anti-CD123 AF700 (R&D, San Diego CA, USA). When necessary for intracellular staining, cells were fixed and permeabilized according to the manufacturer’s instructions (FoxP3/Transcription Factor Staining Buffer, Ebioscience, USA) and stained with the following intracellular antibodies: anti-Ki67 PerCP-Cy5.5, anti-FoxP3 PE and anti-CTLA-4 APC (BD Biosciences, USA). Isotype controls for CD39, CD31, CD25, CD95, Ki67, FoxP3 and CTLA4 were included in each experiment.

We characterized peripheral CD4 T-cells according to the distribution of their maturational subsets [naïve (CD27^+^CD45RA^+^), central memory (CD27^+^CD45RA^−^), effector memory (CD27^−^CD45RA^−^) and TemRA (CD27^−^CD45RA^+^)], also including recent thymic emigrants (RTEs; naïve-CD31^+^) and the expression of activation (HLA-DR), cell-cycle entry (Ki67), senescence (CD57) and prone-to-apoptosis (CD95) markers. We also identified Tregs with classical markers (CD25^hi^FoxP3^+^) and their expression of the mentioned activation markers but also of functional markers (CD39, CTLA-4). We immunophenotyped myeloid dendritic cells (mDCs) as Lin2^−^HLA-DR^+^CD123^−^CD11c^+^ and plasmacytoid dendritic cells (pDCs) as Lin2^−^HLA-DR^+^CD11c^−^CD123^+^.Viable cells were identified using LIVE/DEAD fixable Aqua Blue Dead Cell Stain (Life Technologies, USA). One million cells of each sample were stained, and a minimum of 100,000 events of total lymphocytes and 150,000 dendritic cells were acquired. Flow cytometry was performed on an LSR Fortessa (BD Biosciences, USA). Analysis was performed using FlowJo version 9.3 (TreeStar).

### Statistical analysis

Continuous variables were expressed as medians and interquartile ranges [IQRs] and categorical variables as the number of cases and percentages. Binary logistic regression was used to analyse the potential effect of MVC-cART on the clinical and immunological parameters. Variables with a p value < 0.1 in the univariate analysis were considered in multivariable models. Linear regression analyses were performed to determine factors associated with the magnitude of response (absolute anti-HBs titre). Correlations were assessed using the Spearman’s rho correlation coefficient. A p value of < 0.05 was considered statistically significant. Statistical analysis was performed using SPSS software (version 22; IBM SPSS, Chicago, USA), and graphs were generated using Prism (version 5, GraphPad Software, Inc.).

## Results

### Demographic, clinical and immunological variables associated with MVC-containing cART

Around half of the population (51%) received MVC-cART, consisting of MVC and a boosted protease inhibitor (PI) or MVC and two nucleoside-reverse transcriptase inhibitors (NRTIs). We compared the demographic, clinical and immunological variables between patients receiving MVC-containing cART or MVC-lacking cART at the moment of vaccination (Table 1). The age, CD4^+^/CD8^+^ ratio, time on HIVtreatment, %CD4^+^RTE and %mDCs had *p* values < 0.1 in the univariate analyses and were therefore included in the multivariate analysis. Notably, 19% of the subjects treated with MVC-cART were also receiving NRTIs, whereas 70% of the subjects on MVC-lacking cART were receiving NRTIs. Thus, the absence of NRTIs was highly collinear with the presence of MVC and was not included for adjustment in the multivariate analysis. As is shown, the CD4^+^/CD8^+^ ratio (*p *= 0.086; OR [95% CI], 0.19 [0.03–1.26]) showed a trend toward independent association; however, %CD4^+^RTE (*p *= 0.024; OR [95% CI], 1.20 [1.02–1.41]) and %mDCs (*p *= 0.048; OR [95% CI], 0.16 [0.02–0.98]) were independently associated with MVC-cART.

**Table 1:** Demographic, clinical and immunological variables associated with MVC-containing cART

Demographic, clinical and immunological variables (n = 41)	MVC-containing cART (N = 21)	MVC-lacking cART (N = 20)	Unadjusted *p* value; OR [95% CI]	Adjusted *p* value; OR [95% CI]
Male sex, n (%)	15 (71)	16 (80)	0.525; 0.625 [0.147–2.659]	
Age (years)	36 [31–44]	44 [39–48]	***0.024; 0.892 [0.800***–***0.985]***	0.878; 1.01 [0.87–1.18]
Nadir CD4^+^ T-cell count (cells/mm^3^)	294 [184–412]	264 [206–379]	0.539; 1.001 [0.997–1.005]	
CD4^+^ T-cell count (cells/mm^3^)	703 [565–869]	725 [529–911]	0.775; 1.000 [0.997–1.002]	
CD8^+^ T-cell count (cells/mm^3^)	781 [538–961]	596 [491–829]	0.109; 1.002 [1.000–1.004]	
CD4^+^/CD8^+^ ratio	0.9 [0.6–1.2]	1.1 [0.9–1.6]	***0.084; 0.309 [0.082***–***1.170]***	*0.086; 0.20 [0.03*–*1.26]*
Time since diagnosis (months)	65 [32–212]	132 [67–235]	0.158; 0.995 [0.989–1.002]	
Time on HIV-treatment (months)	46 [33–147]	120 [64–201]	***0.057; 0.992 [0.984***–***1.000]***	0.105; 0.99 [0.97–1.00]
Sexual transmission, n (%)	18 (86)	18 (90)	0.677; 1.500 [0.223–10.077]	
Previous AIDS, n (%)	1 (5)	1 (5)	0.972; 0.950 [0.055–16.293]	
Previous HCV coinfection, n (%)	4 (19)	1 (5)	0.199; 4.471 [0.454–44.011]	
NRTI containing cART, n (%)*	4 (19)	14 (70)	***0.002; 0.101 [0.024***–***0.430]***	
hsCRP (mg/l)	0.8 [0.5–1.1]	0.9 [0.6–1.5]	0.324; 0.612 [0.231–1.623]	
% CD4^+^ naive	44.1 [34.5–55.6]	44.9 [33.7–50.1]	0.829; 0.995 [0.954–1.039]	
% CD4^+^ RTE	78.0 [69.9–82.5]	69.3 [64.3–76.4]	***0.016; 1.116 [1.021***–***1.220]***	***0.024; 1.20 [1.03***–***1.41]***
% CD4^+^ central memory	29.4 [23.3–33.2]	28.3 [22.9–42.5]	0.752; 0.989 [0.926–1.057]	
% CD4^+^ effector memory	20.0 [18.0–27.5]	22.0 [12.9–28.5]	0.968; 0.999 [0.936–1.065]	
% CD4^+^ TemRA	2.4 [1.1–4.3]	1.6 [0.8–4.4]	0.873; 1.017 [0.831–1.243]	
% CD4^+^ HLA-DR^+^	1.8 [1.0–2.0]	1.6 [1.1–2.3]	0.880; 1.066 [0.464–2.451]	
% CD4^+^ Ki67^+^	2.4 [2.0–3.2]	2.2 [2.1–2.6]	0.219; 1.629 [0.748–3.547]	
% CD4^+^ CD57^+^	4.8 [3.5–8.0]	4.47 [2.1–10.8]	0.879; 0.990 [0.873–1.124]	
% CD4^+^ CD95^+^	55.0 [40.7–66.0]	54.4 [44.1–63.9]	0.590; 1.011 [0.971–1.052]	
% CD4^+^ CD25^hi^FoxP3^+^	1.2 [0.9–2.0]	1.5 [1.1–1.7]	0.992; 0.995 [0.358–2.765]	
% CD4^+^ CD25^hi^FoxP3^+^HLA-DR^+^	13.9 [9.9–21.6]	16.1 [12.2–22.1]	0.543; 0.973 [0.889–1.064]	
% CD4^+^ CD25^hi^FoxP3^+^ki67^+^	18.5 [13.5–25.2]	20.1 [17.6–25.0]	0.216; 0.930 [0.830–1.043]	
% CD4^+^ CD25^hi^FoxP3^+^CD39^+^	82.0 [41.7–88.2]	83.7 [81.2–85.1]	0.716; 1.005 [0.980–1.029]	
% CD4^+^ CD25^hi^FoxP3^+^CTLA4^+^	59.0 [46.2–65.2]	57.7 [47.8–69.2]	0.545; 0.986 [0.941–1.032]	
% mDCs	0.5 [0.3–0.8]	0.8 [0.6–1.4]	***0.042; 0.226 [0.054***–***0.949]***	***0.048; 0.16 [0.03***–***0.98]***
% pDCs	0.2 [0.1–0.2]	0.2 [0.1–0.3]	0.529; 0.109 [0.000–108.297]	

Continuous variables are expressed as median values [IQR], and categorical variables are expressed as the number of cases (%). All demographic, clinical and immunological variables with *p* values of < 0.1 in the unadjusted model, except NRTI-containing cART*, were included in the adjusted model and are shown in bolditalics. Hence, age, CD4^+^/CD8^+^ ratio, time on HIV treatment, %CD4^+^RTE and %mDCs were included in the multivariate model (n = 38). Variables with *p* values of < 0.1 are shown in *italics*. Variables with *p* values of < 0.05 in the adjusted model were considered statistically significant and are shown in bolditalic. * The absence of NRTIs was collinear with the presence of MVC

### Relationship between the time of exposure to MVC-containing cART and immunological variables

**Fig. 1:**
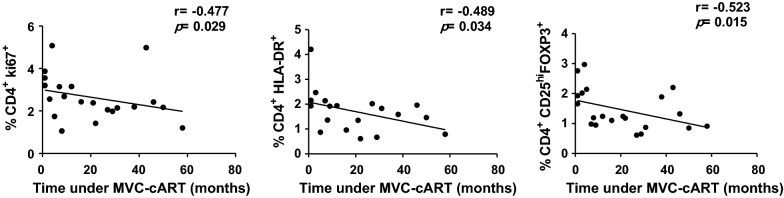
Relationship between the time of exposure to MVC-cART and immunological variables. Only significant correlations between the time of exposure to MVC-cART and immunological variables are represented

Since we observed a high degree of variability in the time of exposure to MVC-cART prior to vaccination (median [IQR], 16 [5–38] months), we explored whether this fact could have affected the immunological variables of the study. This analysis was logically restricted to the MVC-cART group (N = 21) (Additional file 1: Table S1). We found significant negative correlations between the time of exposure to MVC-cART and the %CD4^+^Ki67^+^, the %CD4^+^HLA-DR^+^ and the %CD4^+^CD25^hi^FoxP3^+^ (Fig. 1).

On the other hand, since we expected to find a direct association between MVC-cART and the inflammation-related marker hsCRP, we also explored potential correlations between the five immunological variables affected by MVC-cART in both a direct or a time-dependent way and the levels of hsCRP. hsCRP was only correlated with %CD4^+^RTE (r = − 0.326; *p *= 0.049) and with %CD4^+^HLA-DR^+^ with borderline significance (r = 0.316; *p *= 0.057) (Additional file 2: Figure S1).

### Impact of immunological variables targeted by MVC-cART on the magnitude of the HBV vaccine response

To explore to what extent each of the immunological factors that were affected by MVC-cART could affect the magnitude of the response, we tested potential associations between these five variables and the magnitude of the HBV response. When analysed in the entire cohort (n = 41) (Additional file 3: Table S2), only the %CD4Ki67^+^ showed a negative association with the magnitude of the response, with borderline significance (*p *= 0.053; B [95% CI], − 199.5 [− 401.4 to 2.5]). However, when restricting to the population treated with MVC-cART (n = 21) (Table 2), both the %CD4^+^Ki67^+^ (*p *= 0.027; B [95% CI], − 241.7 [– 452.8 to 30.5]) and the %CD4^+^HLA-DR^+^ (*p *= 0.038; B [95% CI], – 211.2 [– 409.6 to 12.8]) showed significant associations with the anti-HBs titres.

**Table 2:** Relationship between variables affected by MCV-containing cART and the magnitude of the HBV vaccine response

Immunological variables	MVC-cART (n = 21)	Unadjusted p value; B (95% CI)
% CD4^+^ RTE	78.0 [67.0–82.5]	0.599; 6.2 [− 18.0–30.3]
% CD4^+^ HLA-DR^+^	1.8 [1.0–2.0]	***0.038; − 211.2 [− 409.6–12.8]***
% CD4^+^ ki67^+^	2.4 [2.0–3.1]	***0.027; − 241.7 [− 452.8–30.5]***
% CD4^+^ CD25^hi^FoxP3^+^	1.2 [0.9–2.0]	0.889; − 17.0 [− 267.7–233.78]
% mDCs	0.5 [0.3–0.8]	0.272; 196.1 [− 166.3–558.5]

Continuous variables are expressed as median values [IQR]. Linear regression analyses were performed to determine variables associated with the magnitude of response (absolute anti-HBs titre). Variables with *p* values of < 0.1 are shown in *italics*. Variables with *p* values of < 0.05 were considered statistically significant and are shown in bolditalic

## Discussion

We recently observed a beneficial effect of MVC-cART in the HBV vaccine response in a cohort of HIV-infected subjects younger than 50 years old [5]. We report now that HIV-infected subjects on MVC-cART have increased RTE but reduced mDC frequencies prior to vaccination. In addition, a longer time of exposure to MVC-cART was associated with lower frequencies of Tregs and activated and proliferating CD4 T-cells, with proliferating CD4 T-cells being inversely associated with the magnitude of the HBV vaccine response.

In the response to the HBV vaccine, a peptide antigen administered intramuscularly, helper CD4 T-cell function plays a major role [14, 15], and it is well assumed that T-cell exhaustion and senescence related to HIV infection may result in response failure [16]. Antiretroviral treatment improves antigen-specific T-cell responses and recovers the T-cell repertoire [17]. In fact, the duration of cART was associated with the HBV vaccine response [18]. However, the specific effects of different antiretroviral families have been less studied. It is reasonable to expect a negative impact of NRTIs because they favour cellular senescence through inducing accelerated shortening of telomeres in peripheral T-cells [19]. In fact, telomere length has been associated with the response to influenza vaccine in elderly non-HIV-infected subjects [20]. Moreover, we have recently found a better profile in T-cells in subjects on NRTI-lacking regimens regarding cell survival and replicative senescence [21]. On the other hand, there is some controversy about the potential immunological effects of MVC-cART. While some authors have described no effects on inflammatory biomarkers [10, 22], others have comparatively demonstrated their beneficial effects on these markers [6] and on the T-cell immunophenotype [8].

We have now explored the immunological profile associated with MVC-cART in the context of the HBV vaccine response, finding a less activated and proliferative phenotype, a higher contribution of RTEs and a lower frequency of mDC and Treg cells. Notably, HBV vaccine responsiveness has been associated with most of these factors in other cohorts, including decreased activation of T-cells [23], a higher frequency of CD34^+^ precursors [24, 25] and a lower frequency of Tregs [13]. Dendritic cells are being targeted for improvement of HBV vaccine responsiveness [26]. As far as we know, no previous data link proliferative CD4 T-cells with vaccine response in this context. However, it is known that HIV-infected subjects have increased memory CD4 T-cell cycling, which has been proposed to be consequence of the inflammatory environment of HIV infection [27], and a compromised thymic output [28].

Since we found an increase of the RTE frequency along with a reduction of the frequency of proliferating CD4 T-cells associated with MVC-cART, we speculate a potential regenerative capacity for this regimen that requires further research. Indeed, proliferating CD4 T-cells in the absence of thymic output may show limited immunocompetence due to the constriction of TCR diversity [29]. Thus, this regimen could contribute to a potential enrichment of TCR diversity, which would favour the response against vaccine antigens. Along these lines, MVC positively impacted the response to different vaccine antigens in HIV-infected subjects [4, 5].

The effect on Treg cells also deserves discussion. We previously showed a net effect of MVC monotherapy in reducing Tregs in antiretroviral-naïve subjects [11]. In the present cohort of cART-experienced subjects, this reducing effect was dependent on the time of exposure. Notably, both cohorts differed very much, not only because of the presence/absence of treatment but also in age or time from diagnosis, among other factors. Thus, our current results strengthen the hypothesis that MVC exerts immunomodulatory effects through reducing Treg cells. Treg cells are being studied in several immunization models [30–32] because they suppress the proliferation and cytokine secretion of CD4 and CD8 T cells as well as monocytes, dendritic cells and B cells [33, 34]. In fact, Treg cells were found within germinal centres of human lymphoid tissues, suppressing the B cell immunoglobulin class switching needed to mount a proper antibody response [35]. In our cohort, the frequencies of activated and proliferating Treg cells, which could be highly suppressive, were inversely associated with the magnitude of the vaccine response (data not shown).

Importantly, plasma levels of soluble inflammatory markers before vaccination negatively predicted responses to HAV, HBV, and tetanus vaccines in HCV and HIV infection [36]. Moreover, hsCRP levels were a significant predictor of herpes zoster vaccine response in elderly nursing home residents [37]. hsCRP levels were also inversely associated with the magnitude of the vaccine response in our cohort (data not shown), but we failed to observe a direct association between hsCRP levels and MVC-containing cART. However, we cannot exclude a potential effect of MCV-cART on other inflammatory cytokines, as previously reported [6]. Moreover, hsCRP was inversely associated with the frequency of RTEs and positively associated with activated CD4 T-cells, both of which were impacted by MVC-cART, suggesting a potential indirect effect of MVC-cART on the inflammatory state.

As a limitation, the size of our cohort was restricted to the vaccinated population younger than 50 years old, where the effect of MVC-cART on the magnitude of vaccine responsiveness was clear [5]. It is well-known that age limits HBV vaccine responsiveness [38]. Thus, it is reasonable to speculate that the added age-associated immunodeficiency could limit or mask the potential benefits of such antiretroviral therapy on the immunological profile. In this sense, aged people have lower thymic output concomitant with higher peripheral T-cell proliferation [27]. Interestingly, the group on MVC-cART had lower CD4/CD8 ratios, which have been reported to negatively impact the vaccine response [39]. This could be due to the shorter period of treatment in this group, which is critical for CD4/CD8 T-cell ratio normalization [40]. In any case, despite the lower CD4/CD8 ratio, the group on MVC-cART showed better vaccine responsiveness and improved CD4 T-cell profiles. Finally, we cannot discriminate among the particular effects due to the presence of MVC or to the absence of NRTIs in the cART, and thus, we can only draw conclusions about the beneficial effects of such combined therapy. Similar combined therapies are being explored in the clinical setting in an attempt to reduce toxicities and to improve immune reconstitution [41].

## Conclusion

The beneficial effect of MVC-cART in the HBV vaccine response in subjects below 50 years old could be mediated at least partially by its reducing effect on the frequencies of activated and proliferating CD4 T-cells prior to vaccination. This fact could be related with a potential regenerative capacity of such therapy and deserves further research due to its relevance in the search for novel therapeutic targets that could improve immune function and vaccine responsiveness in HIV-infected subjects.

## Additional files

**Additional file 1: Table S1.** Relationship between the time of exposure to MVC-containing cART and immunological variables.

**Additional file 2: Figure S1.** Associations between hsCRP and T-cell immunological variables affected by MVC-cART. Only significant correlations between hsCRP and T-cell immunological variables are represented.

**Additional file 3: Table S2.** Relationship between variables modified by MCV-containing cART and the magnitude of the HBV vaccine response in the whole cohort.

## Abbreviations

cART: combined antiretroviral therapy
DC: dendritic cell
ELISA: enzyme-linked immunosorbent assay
HAV: hepatitis A virus
HBV: hepatitis B virus
HCV: hepatitis C virus
HIV: human immunodeficiency virus
hsCRP: highly sensitive C-reactive protein
mDC: myeloid-DC
MVC: maraviroc
MVC-cART: maraviroc-containing combined antiretroviral therapy
NRTI: nucleoside reverse-transcriptase inhibitors
PBMCs: peripheral blood mononuclear cells
pDC: plamacytoid dendritic cells
PI: protease inhibitor
RTE: recent thymic emigrants
Treg: regulatory T-cells
