# Supplementary material for: Improved CD4 T cell profile in HIV-infected subjects on maraviroc-containing therapy is associated with better responsiveness to HBV vaccination
Source: J Transl Med. 2018 Aug 29;16:238. doi: 10.1186/s12967-018-1617-1 (PMC6116502; doi:10.1186/s12967-018-1617-1)
Supplement: Supplementary file 1 — Additional file 1: Table S1. Relationship between the time of exposure to MVC-containing cART and immunological variables. [file 12967_2018_1617_MOESM1_ESM.docx]

**Supplementary Table 1. Relationship between the time of exposure to MVC-containing cART and immunological variables.**

| **Immunological variables (n=21)** | **r** | **p** |
| --- | --- | --- |
| **hsCRP (mg/L)** | 0.003 | 0.989 |
| **% CD4^+^ Naive** | 0.185 | 0.434 |
| **% CD4^+^ RTE** | -0.161 | 0.497 |
| **% CD4^+^ Central Memory** | -0.151 | 0.526 |
| **% CD4^+^ Effector Memory** | -0.085 | 0.723 |
| **% CD4^+^ TemRA** | -0.087 | 0.714 |
| **% CD4^+^ HLA-DR^+^** | -0.489 | **0.034** |
| **% CD4^+^ Ki67^+^** | -0.477 | **0.029** |
| **% CD4^+^ CD57^+^** | 0.110 | 0.663 |
| **% CD4^+^ CD95^+^** | -0.170 | 0.460 |
| **% CD4^+^ CD25^hi^FoxP3^+^** | -0.523 | **0.015** |
| **% CD4^+^ CD25^hi^FoxP3^+^HLA-DR^+^** | -0.126 | 0.586 |
| **% CD4^+^ CD25^hi^FoxP3^+^ki67^+^** | -0.318 | 0.172 |
| **% CD4^+^ CD25^hi^FoxP3^+^CD39^+^** | 0.116 | 0.617 |
| **% CD4^+^ CD25^hi^FoxP3^+^CTLA4^+^** | 0.226 | 0.324 |
| **% mDCs** | -0.133 | 0.567 |
| **% pDCs** | 0.105 | 0.650 |

Correlations were assessed using Spearman's rho correlation coefficient. Variables with *p* values of <0.05 were considered statistically significant and are shown in bold.
