# Supplementary figures and images for: Improved CD4 T cell profile in HIV-infected subjects on maraviroc-containing therapy is associated with better responsiveness to HBV vaccination
Source: J Transl Med. 2018 Aug 29;16:238. doi: 10.1186/s12967-018-1617-1 (PMC6116502; doi:10.1186/s12967-018-1617-1)

## Slide 1
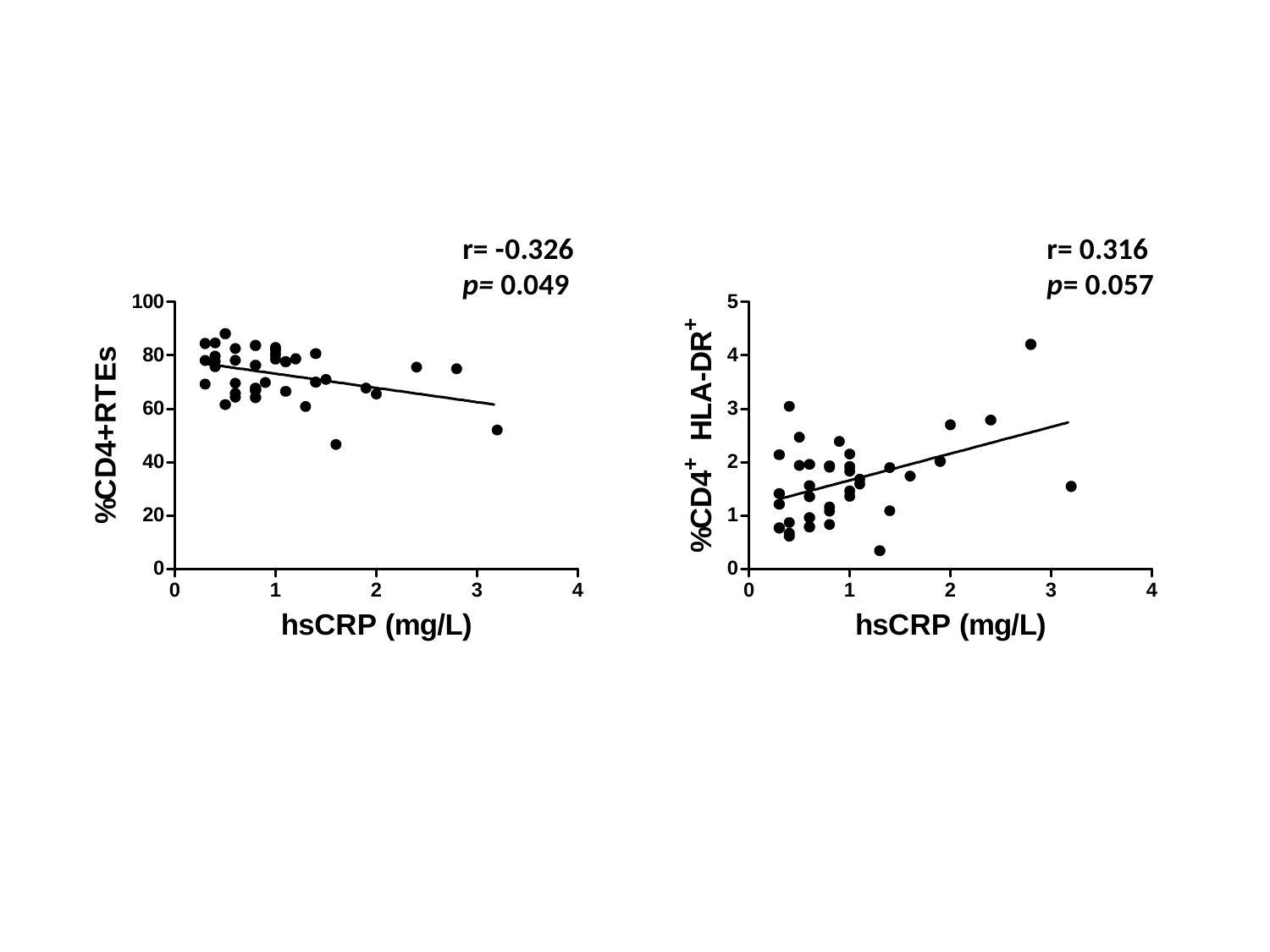

Supplement: Supplementary file 2 — Additional file 2: Figure S1. Associations between hsCRP and T-cell immunological variables affected by MVC-cART. Only significant correlations between hsCRP and T-cell immunological variables are represented. [file 12967_2018_1617_MOESM2_ESM.pptx]
