# Supplementary material for: Improved CD4 T cell profile in HIV-infected subjects on maraviroc-containing therapy is associated with better responsiveness to HBV vaccination
Source: J Transl Med. 2018 Aug 29;16:238. doi: 10.1186/s12967-018-1617-1 (PMC6116502; doi:10.1186/s12967-018-1617-1)
Supplement: Supplementary file 3 — Additional file 3: Table S2. Relationship between variables modified by MCV-containing cART and the magnitude of the HBV vaccine response in the whole cohort. [file 12967_2018_1617_MOESM3_ESM.docx]

**Supplementary Table 2. Relationship between variables modified by MCV-containing cART and the magnitude of the HBV vaccine response in the whole cohort.**

| **Immunological variables** | **All cohort**  **(n=41)** | **Unadjusted P value; B (95% CI)** |
| --- | --- | --- |
| **% CD4^+^ RTE** | 75.6 [66.5-79.8] | 0.204; 9.2 [-5.2­-23.7] |
| **% CD4^+^ HLA-DR^+^** | 1.7 [1.1-2.0] | 0.161; -128.1 [-309.6-53.4] |
| **% CD4^+^ ki67^+^** | 2.3 [2.1-2.7] | *0.053;* -199.5 [-401.4-2.5] |
| **% CD4^+^ CD25^hi^FoxP3^+^** | 1.3 [1.0-1.8] | 0.565; -64.7 [-290.3-160.9] |
| **% mDCs** | 0.7 [0.4-1.1] | 0.477; -85.4 [-325.9-155.1] |

Continuous variables are expressed as median values [IQR]. Linear regression analyses were performed to determine variables associated with the magnitude of response (absolute anti-HBs titre). Variables with *p* values of <0.1 are shown in *italics*.
